# Supplementary material for: A review and meta-analysis of the enemy release hypothesis in plant–herbivorous insect systems
Source: PeerJ. 2016 Dec 21;4:e2778. doi: 10.7717/peerj.2778 (PMC5180588; doi:10.7717/peerj.2778)
Supplement: Supplemental Information 1 [file peerj-04-2778-s001.doc]

**PRISMA 2009 Flow Diagram**

**Screening**

**Included**

**Eligibility**

**Identification**

Records identified through database searching
(n = 250 )

Additional records identified through other sources
(n = 3 )

Records screened
(n = 253)

Records excluded
(n = 200)

Full-text articles assessed for eligibility
(n = 53)

Full-text articles excluded, with reasons
(n = 9)

Studies included in qualitative synthesis
(n = 44)

Data sets included in quantitative synthesis (meta-analysis)
(n = 68)
